# Integrative Analysis Revealed LINC00847 as a Potential Target of Tumor Immunotherapy

**DOI:** 10.1007/s12010-023-04387-z

**Published:** 2023-03-03

**Authors:** Xiujuan Chen, Le Zhang

**Affiliations:** Center for Reproductive Medicine, The Affiliated Hospital of Inner Mongolia Medical University, 1 Tong Dao Street, Huimin District, 010050 Hohhot, Inner Mongolia China

**Keywords:** Lung adenocarcinoma, Immune, Immunotherapy, LINC00847, Survival

## Abstract

Lung cancer is the second most commonly diagnosed cancer and the leading cause of cancer-related death. Lung adenocarcinoma (LUAD) is the most common form of lung cancer and has a low 5-year survival rate. Therefore, much more research is needed to identify cancer biomarkers, promote biomarker-driven therapy and improve treatment outcomes. LncRNAs have been reported to participate in various physiological and pathological processes, especially in cancer, and thus have attracted much attention. In this study, lncRNAs were screened from the single-cell RNA-seq dataset CancerSEA. Among them, four lncRNAs (HCG18, NNT-AS1 and LINC00847 and CYTOR) were closely associated with the prognosis of LUAD patients according to Kaplan–Meier analysis. Further study explored the correlations between these four lncRNAs and immune cell infiltration in cancer. In LUAD, LINC00847 was positively correlated with the immune infiltration of B cells, CD8 T cells, and dendritic cells. LINC00847 decreased the expression of PD-L1, immune checkpoint blockade (ICB) immunotherapy-related gene, which suggests that LINC00847 is a potential new target for tumor immunotherapy.

**Supplementary Information:**

The online version contains supplementary material available at 10.1007/s12010-023-04387-z.

## Introduction

Cancer is a major burden on human health in every country. According to the International Agency for Research on Cancer, among 36 cancers in 185 countries, there were an estimated 19.3 million new cancer cases and almost 10.0 million cancer-related deaths in 2020 [1]. Lung cancer is the second most commonly diagnosed cancer and the leading cause of cancer-related death [1]. There is expected to be 350 deaths per day in the United States from lung cancer in 2022 [2]. In most countries, the 5-year relative survival rate of lung cancer was only 10-20% from 2010 to 2014 [3]. Non-small cell lung cancer (NSCLC) is the most common subtype of lung cancer, accounting for approximately 85% of lung cancer cases [4]. NSCLC is subdivided into lung adenocarcinoma (LUAD) and lung squamous cell carcinoma (LUSC) [5]. Despite improved disease awareness, treatment options, and outcomes for lung cancer, survival rates for lung cancer remain unsatisfactory. Many more efforts into identifying cancer biomarkers, promoting biomarker-driven therapy and facilitating drug development are needed to improve the treatment and prognosis of lung cancer.

Long noncoding RNAs (lncRNAs) are a type of noncoding RNA (ncRNA) with a length of more than 200 nucleotides [6]. lncRNAs have already been identified as key regulators in physiological processes. For instance, existing studies have provided insights into the physiological functions of lncRNAs, including organ development, immunity and organismal viability, based on lncRNA knockout mice [7]. lncRNAs also participate in various pathological processes, such as those related to central nervous system disorders [8], diabetes [9], cardiovascular diseases [10] and cancer. Owing to their crucial roles in the initiation and progression of cancer, lncRNAs have been a research hotspot attracting much attention.

Immunotherapy is a type of treatment that activates or inactivates the immune response to fight disease. Immune checkpoint inhibitors (ICIs), a well-known type of immunotherapy, block the “brakes” of the immune system to activate the immune response by targeting PD-1, CTLA-4, LAG-3 and TIM-3 [11]. The PD-1/PD-L1 axis inhibits T-cell activation, proliferation, survival and cytotoxic secretion within cancer cells [12]. Importantly, Wang and colleagues found that PD-1 was expressed across a broad range of tumor cells and that overexpressing/silencing PD-1 or PD-L1 could inhibit/promote tumor cell proliferation and tumor growth in vitro and in vivo [13]. Their study uncovered the potential tumor suppressor role of PD-1/PD-L1, providing a potential biomarker for optimal cancer immunotherapeutic treatment [13]. Our study screened lncRNAs from the single-cell RNA-seq dataset CancerSEA. Then, we revealed their relevant characteristics, including sublocation, conservation, methylation pattern, and correlation with cancer hallmarks, through lncSEA dataset analysis. Kaplan–Meier analysis indicated that four lncRNAs (HCG18, NNT-AS1 and LINC00847 and CYTOR) were closely associated with the prognosis of LUAD patients. Furthermore, we explored the correlation between these four lncRNAs and immune cell infiltration in cancer. In LUAD, LINC00847 was positively correlated with the immune infiltration of B cells, CD8 T cells, and dendritic cells. By using western blotting, we found that LINC00847 decreased the expression of immune checkpoint blockade (ICB) immunotherapy-related gene PD-L1. This study proved that LINC00847 might be a potential new target for tumor immunotherapy.

## Materials and Methods

### Identification of the Expressed lncRNAs

The expression profile of lncRNAs was obtained from CancerSEA (http://biocc.hrbmu.edu.cn/CancerSEA/). TBtools was employed to identify the intersecting lncRNAs in seven different types of cancer tissues. Ninety-one lncRNAs overlapped in seven different types of cancer. Gene annotation was performed with Ensembl (https://asia.ensembl.org/index.html). Then, a gene list containing 75 lncRNAs with annotations was submitted to analyze their relevant characteristics through the lncSEA dataset (http://bio.liclab.net/LncSEA/index.php).

### Kaplan–Meier Survival Analyses

The effects of the lncRNAs on OS and PFS were assessed via the Kaplan–Meier Plotter website (http://kmplot.com/analysis/index.php?p=background). Kaplan–Meier survival analyses of patients for whom OS data were available in the TCGA dataset were performed using the Oncolnc website (http://www.oncolnc.org/).

### Tumor‑Infiltrating Immune Cells

ImmLnc is a web-based resource for immune lncRNAs in cancer (http://bio-bigdata.hrbmu.edu.cn/ImmLnc). The correlation of the expression of four lncRNAs with six tumor-infiltrating immune cells, including B cells, CD4 T cells, CD8 T cells, dendritic cells, macrophages and neutrophils, was performed to determine whether the four lncRNAs may serve as crucial regulators in immune infiltration in cancer.

### Cell Culture

The immortalized human bronchial epithelial cell line BEAS-2B and NSCLC cell lines, including NCI-H1299, NCI-H1975, A549, HCC827 and PC9, were obtained from the American Type Culture Collection (ATCC). All cells were cultured according to the manufacturer’s instructions. BEAS-2B was grown in BEGM (Lonza/Clonetics) with all the additives provided with the BEGM kit except for GA­1000 (gentamycin-amphotericin B mix). NSCLC cell lines were cultured in DMEM (Invitrogen) supplemented with 10% FBS (Gibco) and 100 U/ml penicillin/streptomycin (Invitrogen).

### Plasmids and Transfection

The LINC00847 expression plasmids were generated by PCR subcloning of the human LINC00847 coding sequence into the pLVX-IRES plasmid. A549 and H1975 cells were transfected with Lipofectamine 3000 (Thermo Scientific) according to the manufacturer’s instructions.

### Western Blotting Analysis

Proteins were separated on denaturing SDS‒PAGE gels and transferred onto PVDF membranes. Membranes were blocked with 5% nonfat milk for 1 h at room temperature and incubated overnight at 4 °C with anti-PD-L1 antibody (13,684, CST), anti-P84 antibody (10920-1-AP, Proteintech), and anti-β-actin (K200058M, Solarbio). Then, the membranes were washed and incubated for 1 h with an HRP-conjugated secondary antibody. An ECL chemiluminescence system (Tanon-4800 Multi) was employed for the detection of proteins.

### Statistical Analysis

The correlation of lncRNAs with six tumor-infiltrating immune cells and immune checkpoint molecules was examined by Spearman’s correlation and the estimated statistical significance. The association between lncRNAs and the prognosis of LUAD patients was evaluated by Kaplan–Meier survival curves with the log-rank test. All statistical analyses were performed with the SPSS 20.0 software package (IBM, Chicago, IL, USA). Two-tailed Student’s t test or a chi-squared test was used to determine the significance of the differences. All error bars represent the mean ± SEM. P values are shown as follows: not significant (ns); P value < 0.05 (*); P value < 0.01 (**); P value < 0.001 (***).

## Results

### Analysis of lncRNA Expression Profiles Through Single-Cell Sequencing

**Fig. 1:**
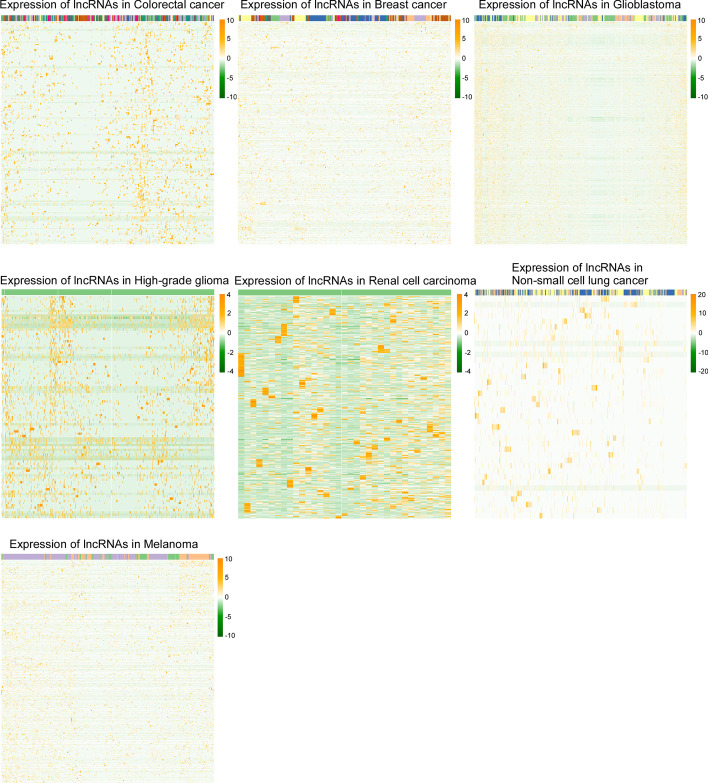
lncRNA expression profiles obtained by analyzing a single-cell sequence dataset. The expression profiles of lncRNAs in seven different types of cancer tissues are shown in a heatmap by downloading from CancerSEA; the hierarchically clustered lncRNAs and clustered cells are depicted as different rows and columns, respectively

CancerSEA(http://biocc.hrbmu.edu.cn/CancerSEA/) provides a method to uncover the distinct functional states of cancer cells at single-cell resolution. We downloaded expression and functional state profiles based on single-cell RNA-seq from seven different types of cancer tissues, including colorectal cancer, breast cancer, glioblastoma, high-grade glioma, renal cell carcinoma, non-small cell lung cancer, and melanoma tissues. The expression profile of lncRNAs is shown in a heatmap, in which the hierarchically clustered lncRNAs and clustered cells are depicted as different rows and columns, respectively (Fig. 1).

### Identification of the Intersection of lncRNAs

**Fig. 2:**
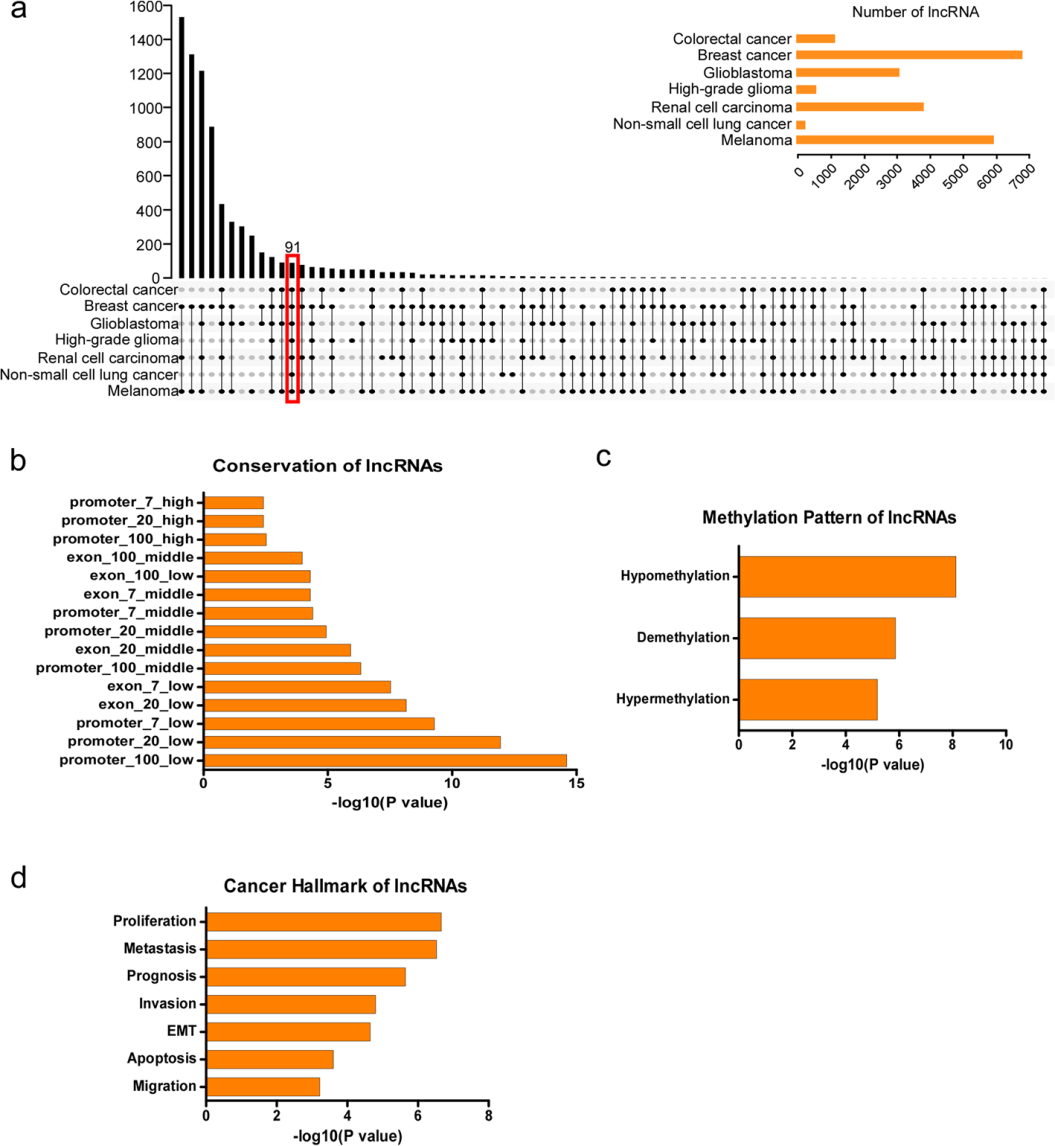
Identification of the intersecting lncRNAs among seven types of cancer. **a** TBtools was used to identify the intersecting lncRNAs among seven different types of cancer tissues. Gene annotation was performed with the Ensembl dataset. **b-d** Relevant characteristics of lncRNAs, such as conservation, methylation pattern and cancer hallmark correlation, were analyzed through the lncSEA dataset

To identify the intersection of lncRNAs in the seven different types of cancer tissues described above, we generated an UpSet plot with TBtools. Ninety-one lncRNAs overlapped in seven different types of cancer (Fig. 2a). Then, we converted the Ensembl gene IDs of 91 lncRNAs to official gene symbols via DAVID (https://david.ncifcrf.gov/home.jsp) and lncSEA (http://bio.liclab.net/LncSEA/index.php). We found that 75 lncRNAs had a gene annotation in Ensembl (https://asia.ensembl.org/index.html), and 16 lncRNAs were novel transcripts (Supplementary Table 1). Some of the imprinted lncRNAs responsible for cancer initiation and development have been identified; these include NEAT1 [14], MALAT1 [15], Xist [16], and ZEB1-AS1 [17]. The 16 screened lncRNAs without gene annotation may also play a critical role in cancer progression. Further studies are needed to validate and test this speculation. Then, we submitted a gene list containing these 75 lncRNAs to analyze their relevant characteristics through the lncSEA dataset (http://bio.liclab.net/LncSEA/index.php). We discovered that these lncRNAs were mainly located in the cytoplasm, nucleus, ribosome, and exosome (Supplementary Table 2). The conservation degree of lncRNAs varied between vertebrates and mammals (Fig. 2b, Supplementary Table 3). The methylation pattern is only reported for 6 lncRNAs. KCNQ1OT1, TP53TG1, MALAT1 and ZEB1-AS1 are reported to be hypomethylated; KCNQ1OT1, NEAT1 and DLEU2 are reported to be demethylated; KCNQ1OT1, DLEU2 and TP53TG1 remains hypermethylated (Fig. 2c, Supplementary Table 4). As for association with cancer hallmarks, these lncRNAs are correlated with proliferation, metastasis, prognosis, EMT, apoptosis, invasion and migration (Fig. 2d, Supplementary Table 5).

### LncRNAs Correlated with the Prognosis of LUAD Patients

**Fig. 3:**
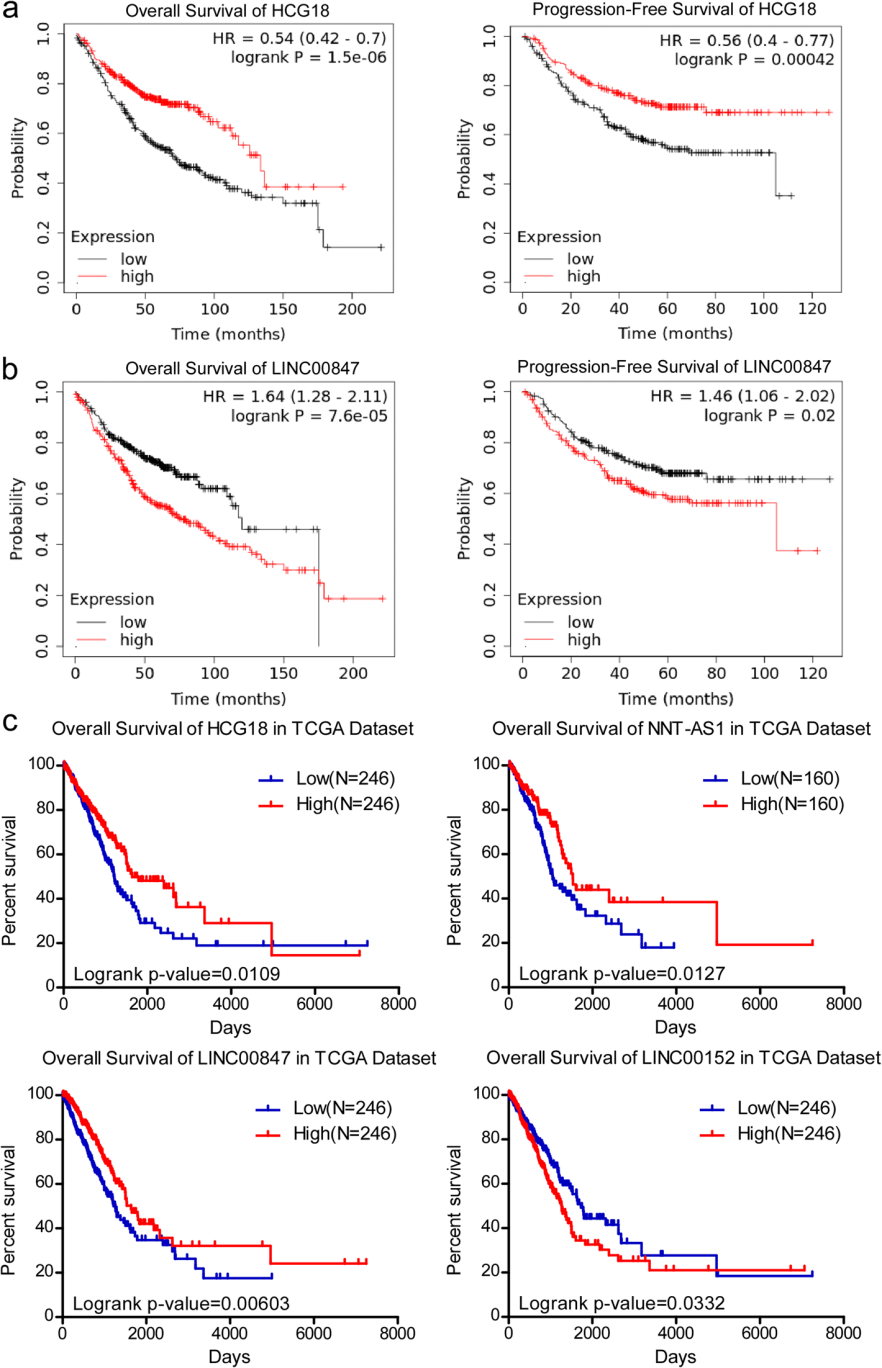
Kaplan–Meier analysis of the OS and PFS of LUAD patients.** a**,** b** Kaplan‒Meier survival analysis of HCG18 and LINC00847 based on data collected from Kaplan–Meier Plotter. **c** Kaplan–Meier analysis of OS based on data for the four lncRNAs available in TCGA dataset through OncoLnc website

To uncover the lncRNAs that could predict the prognosis of LUAD patients, we initially analyzed the overall survival (OS) and progression-free survival (PFS) of groups sorted according to the expression of these lncRNAs with the Kaplan–Meier Plotter website (http://kmplot.com/analysis/index.php?p=background). The results indicated that the OS and PFS of patients with high HCG18 expression were better than those with low HCG18 expression (OS: log rank test, *P* < 0.001; PFS: log rank test, *P* < 0.001) (Fig. 3a). High LINC00847 expression was correlated with reduced OS and PFS in patients (OS: log rank test, *P* < 0.001; PFS: log rank test, *P* = 0.02) (Fig. 3b). Furthermore, we analyzed lncRNA expression profiles for whom OS data were available in the TCGA dataset through the Oncolnc website (http://www.oncolnc.org/). We found that high expression of HCG18, NNT-AS1 and LINC00847 was associated with better OS (log rank test, *p* = 0.0109; *p* = 0.0127; *p* = 0.0060, respectively), while high expression of CYTOR (LINC00152) was correlated with shorter OS (log rank test, *p* = 0.0332) (Fig. 3c). Controversially, the association between LINC00847 and OS in different datasets was the opposite. It was reported that LINC00847 promotes non-small cell lung cancer progression by targeting the miR-147a/IFITM1 axis [18]. In hepatocellular carcinoma, LINC00847 accelerates cancer progression by acting as a sponge of miR-99a to induce E2F2 expression [19]. Given these studies, LINC00847 may function as an oncogene in cancer progression.

### Correlation of lncRNAs with Immune Cell Infiltration

**Fig. 4:**
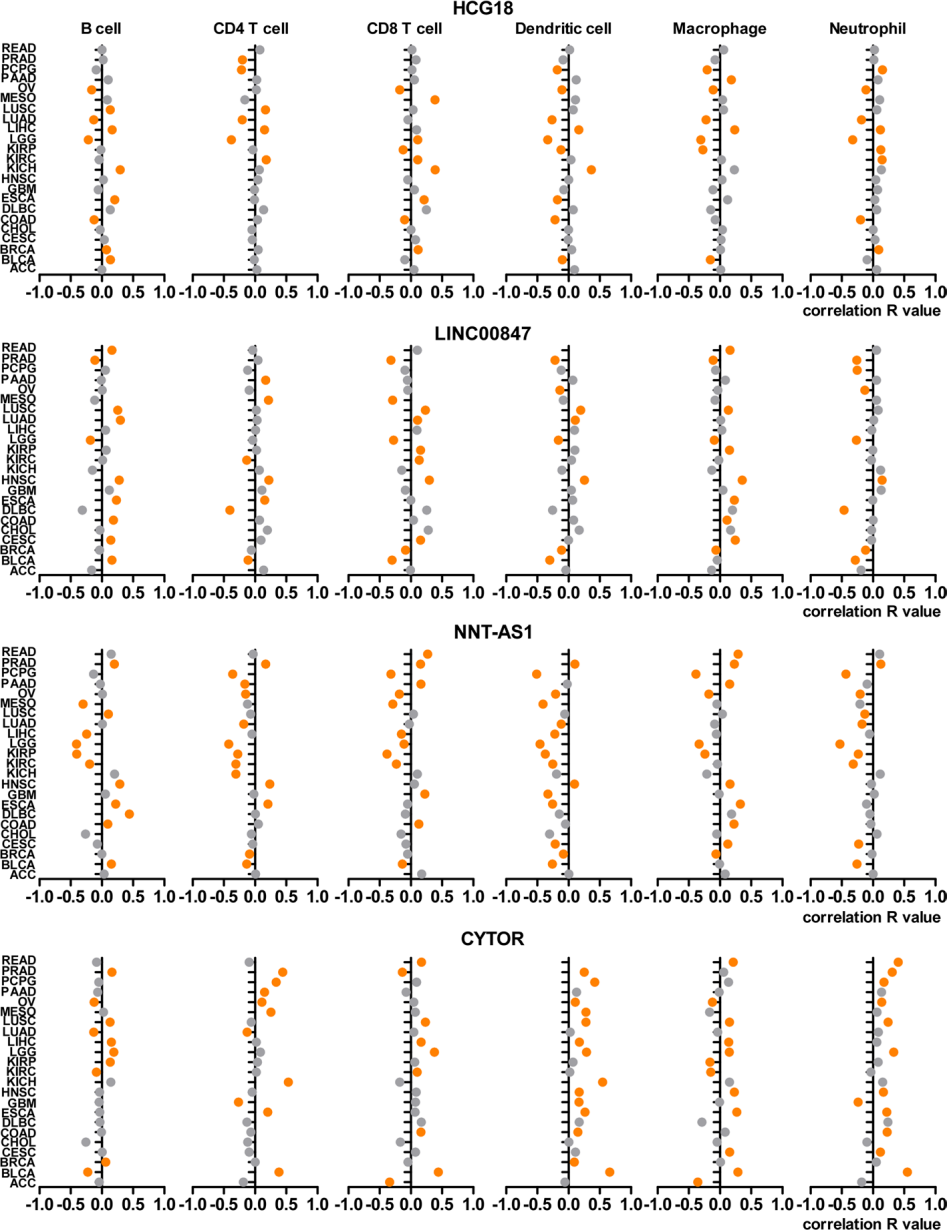
Association between lncRNAs and immune cell infiltration. Association between the four lncRNAs (HCG18, LINC00847, NNT-AS1 and CYTOR) and six types of tumor-infiltrating immune cells, including B cells, CD4 T cells, CD8 T cells, dendritic cells, macrophages and neutrophils. The Y axis shows the names of the cancer type. X-axis label represents correlation R value. The orange dot indicates that p value is less than 0.05 and is statistically significant

Previously, we screened four lncRNAs (HCG18, NNT-AS1, LINC00847 and CYTOR (LINC00152)) closely related to the prognosis of LUAD patients. Existing evidence has revealed their roles in the proliferation, migration, and angiogenesis of non-small cell lung cancer [20–24], hepatocellular carcinoma [25], kidney cancer [26], colon cancer [27], colorectal cancer [28, 29], breast cancer [30, 31] and myeloma [32]. However, research on their association with immune reactions remains limited. Here, we explored the correlation between these four lncRNAs and immune cell infiltration in cancer. lncRNA-immune cell type correlation data were downloaded from the ImmLnc website (http://bio-bigdata.hrbmu.edu.cn/ImmLnc). We analyzed six tumor-infiltrating immune cells, including B cells, CD4 T cells, CD8 T cells, dendritic cells, macrophages and neutrophils. The results showed that these four lncRNAs were associated with immune cell infiltration in various types of cancer (Fig. 4). In LUAD, HCG18 was negatively correlated with the infiltration of B cells (*r *=  -0.13; *p* = 0.003), CD4 T cell (*r* = -0.21; *p* < 0.001), neutrophil (*r* = -0.18; *p* < 0.001), macrophage (*r* = -0.22; *p* < 0.001), and dendritic cells (*r* = -0.27; *p* < 0.001). Similarly, NNT-AS1 was negatively correlated with the infiltration of CD4 T cell (*r *= -0.18; *p* < 0.001), neutrophil (*r *= -0.18; *p* < 0.001), and dendritic cells (*r* = -0.12; *p* = 0.007). CYTOR was negatively correlated with B cells (*r* = -0.13; *p* = 0.003) and CD4 T cell (*r* = -0.13; *p* = 0.004). In contrast, LINC00847 was positively correlated with the infiltration of B cells (*r* = 0.30; *p* < 0.001), CD8 T cells (*r* = 0.11; *p* = 0.016), and dendritic cells (*r* = 0.11; *p* = 0.015) in LUAD.

### LINC00847 Regulates Immune Checkpoint Blockade Gene

**Fig. 5:**
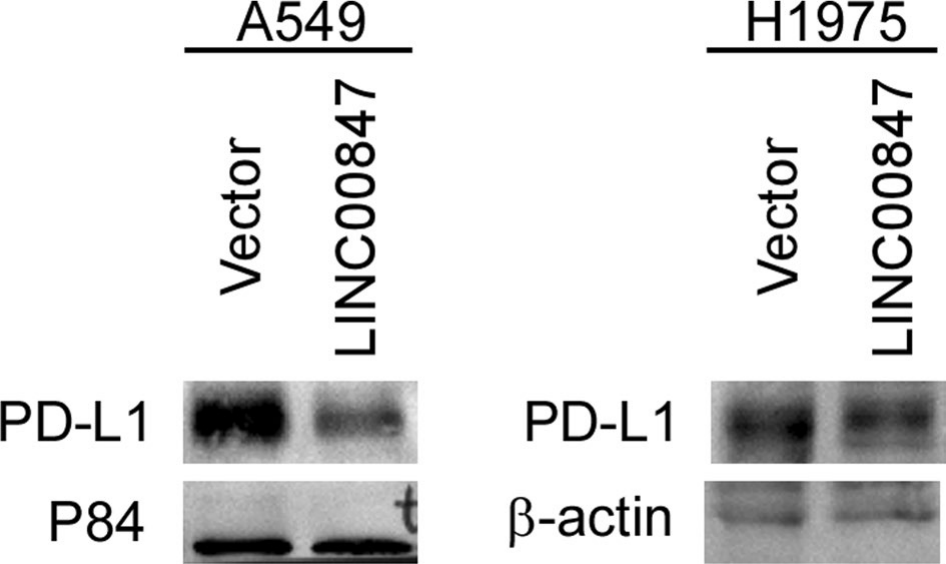
LINC00847 regulates ICB-related gene expression. Western blotting was used to assess the expression of the ICB immunotherapy-related gene PD-L1

It was previously reported that LINC00847 promotes proliferation, invasion and migration. Our research found that LINC00847 was related to immune infiltration. However, there is no previous research associated with this finding. Therefore, we performed western blotting to further verify the results. The LUAD cell lines A549 and H1975 were transfected with pLVX-IRES-vector or pLVX-IRES-LINC00847 plasmids by using Lipofectamine 3000 according to the manufacturer’s instructions. The cells were harvested 48 h posttransfection. Western blotting was used to examine the protein expression level of PD-L1 (Fig. 5). The data showed that overexpression of LINC00847 could significantly downregulate PD-L1 protein expression. LINC00847 decreased the expression of PD-L1, immune checkpoint blockade (ICB) immunotherapy-related gene, indicating that it might be a potential new target for tumor immunotherapy.

## Discussion

Single-cell sequencing could provide information for identifying gene expression changes responsible for altering cellular function, regional specificity, and stage of differentiation by analyzing cells on an individual level. It is a powerful approach to distinguish key contributors ignored at the population level. By using expression and functional state profile-based single-cell RNA-seq data from seven different types of cancer tissues, we identified 91 lncRNAs overlapping in these seven types of cancer. Intriguingly, there were 75 lncRNAs with gene annotation information in Ensembl and 16 lncRNAs described as novel transcripts. Among the 75 annotated lncRNAs, we discovered many well-studied lncRNAs responsible for cancer initiation and development. For instance, MALAT1 regulates cancer cell proliferation [33, 34], differentiation [35], metastasis [36, 37] and chemoresistance [38] through various mechanisms, such as overexpression, translocation and amplifications. Existing evidence has shown that NEAT1 acts as an oncogene in most types of cancer (non-small cell lung cancer, pancreatic cancer, osteosarcoma, renal cell carcinoma and so on), while in acute promyelocytic leukemia, it functions as a tumor suppressor [39, 40]. Xist is reported to be dysregulated in various types of cancer, which influences different hallmarks of cancer [41–44]. Moreover, there are many lncRNAs (ZEB1-AS1 [45, 46], NORAD [47], LINC00511 [48, 49], LINC00667 [50, 51], etc.) that exert regulatory and functional roles in cancer progression and have potential prognostic value. These results confirm that lncRNAs identified by this method are crucial regulators involved in cancer. Based on this, we propose that 16 unannotated lncRNAs may also be closely associated with cancer progression, and much more research is needed to validate and test this hypothesis.

Analyzing existing datasets is very useful for predicting the survival and prognosis of cancer patients in order to compare potentially effective markers and guide treatment decisions. We discovered that the expression of HCG18 was positively correlated with the OS and PFS of LUAD patients, while LINC00847 was inversely associated with OS and PFS by Kaplan–Meier Plotter dataset analysis. In addition, in a cohort of LUAD patients from the TCGA, upregulation of HCG18, NNT-AS1 and LINC00847 was positively correlated with OS, while upregulation of CYTOR (LINC00152) was negatively associated with OS. Controversially, the direction of association between LINC00847 and OS in different datasets was the opposite. The possible reasons might be the source of population and the size of sample are different (TCGA:n = 492; Kaplan–Meier Plotter: n = 672). However, based on research of LINC00847 in cancer, it might be an oncogene related to cancer progression. Combining survival and prognosis studies with existing studies could better validate the roles of lncRNAs in cancer and provide new insight for the diagnosis and treatment of cancer.

Next, we focused on the four screened lncRNAs (HCG18, NNT-AS1, LINC00847 and CYTOR) that were closely related to the prognosis of LUAD patients. It was reported that HCG18 accelerates the progression of LUAD by acting as a sponge of miR-34a-5p to upregulate the expression of HMMR [23]. LINC00847 induces by E2F1 facilitates non-small cell lung cancer progression by targeting the miR-147a/IFITM1 axis [18]. NNT-AS1 has been reported to function as a sponge of miRNA (miR-22 and miR-3666) that regulates the progression of lung cancer [21, 22, 24]. CYTOR promotes LUAD proliferation by interacting with EZH2 and repressing IL24 expression [20]. However, the relationship between these lncRNAs and the immune response is unclear. We explored the correlation between these four lncRNAs and immune cell infiltration in cancer by evaluating data from ImmLnc. Importantly, we found that LINC00847 was positively correlated with the immune infiltration of B cells, CD8 T cells, and dendritic cells in LUAD. Given that there is no sufficient experimental evidence to prove the association of these lncRNAs with the immune response, we performed western blotting to further verify the results. The results revealed that overexpression of LINC00847 led to significant downregulation of PD-L1 at the protein level. Our study indicated that LINC00847 might be a potential new target for tumor immunotherapy.

## Conclusion

This study revealed that LINC00847 was associated with prognosis and immune infiltration and confirmed that LINC00847 decreased the expression of the immunotherapy-related gene PD-L1, which might prove that LINC00847 is a potential new target for tumor immunotherapy.

### Supplementary Information

Below is the link to the electronic supplementary material.

ESM 1 (XLSX 11.8 KB)

ESM 2 (XLSX 12 KB)

ESM 3 (XLSX 12.7 KB)

ESM 4 (XLSX 10.3 KB)

ESM 5 (XLSX 10.8 KB)

## Data Availability

All data supporting the conclusions of this article are included within the article.
